# Biomechanical Influence of Placement Angle and Loading Direction of Orthodontic Miniscrews on Orthotropic Mandible

**DOI:** 10.3390/ma18214963

**Published:** 2025-10-30

**Authors:** Yu-Ching Li, Jiun-Ren Hwang, Chin-Ping Fung, Chen-Yuan Chung

**Affiliations:** 1 Department of Mechanical Engineering, National Central University, Taoyuan 320317, Taiwan; liyuchingbigboss@gmail.com (Y.-C.L.); cychung@ncu.edu.tw (C.-Y.C.); 2 Department of Mechanical Engineering, Asia Eastern University of Science and Technology, New Taipei City 220303, Taiwan; cpfung@mail.aeust.edu.tw

**Keywords:** miniscrews, oblique insertion, finite element analysis (FEA), isotropic, orthotropic

## Abstract

FEA of orthodontic miniscrews has predominantly assumed isotropic, homogeneous bone, neglecting directional variations in mechanical properties. This study investigated the biomechanical behavior of miniscrews under different insertion angles and loading directions using both isotropic and orthotropic mandibular bone models. The results indicated that isotropic modeling may underestimate miniscrew displacement and associated instability, whereas orthotropic material properties better reflect the true mechanical response of bone. Oblique insertion at 60° (U60°) led to higher strain and greater variability, which may compromise osseointegration; aligning the loading direction parallel to the insertion plane is therefore recommended when oblique placement is unavoidable. Screw thread design had minimal influence on displacement, von Mises stress, or bone strain during vertical insertion. Stress and strain distributions exhibited symmetry, suggesting that analyzing partial loading directions can predict the overall biomechanical response. All predicted values remained below bone and material strength limits, confirming the mechanical safety of the current miniscrew design under a 2 N load. Implant failure is likely attributable to poor osseointegration or inflammation rather than structural limitations.

## 1. Introduction

In orthodontic treatment, the magnitude and direction of the forces required for tooth movement are largely determined by the clinician’s experience and expertise. Before the widespread application of miniscrews, teeth themselves were commonly used as anchorage units. However, this approach has a significant drawback: the teeth serving as anchors may move due to reaction forces, causing unintended tooth displacement. To mitigate this issue, extraoral headgear devices were traditionally used to stabilize the anchorage teeth and prevent unwanted movement. Nevertheless, such devices are inconvenient, interfere with daily activities, and negatively affect facial esthetics, which often result in patient dissatisfaction [1,2].

With the rapid development of orthodontics, a variety of temporary anchorage devices have been introduced, among which miniscrews have become the most popular and effective option. They are easy to implant, offer flexible placement options, are cost-effective, and can be applied immediately without waiting for osseointegration, thereby significantly reducing overall treatment time [3,4].

In clinical practice, a power chain is commonly used to connect the miniscrew and the teeth at both ends, applying a force of 2 to 3 N (approximately 200–300 g) for various types of tooth movement [5]. Furthermore, since the direction of tooth movement varies among clinical cases, miniscrews implanted in the same position may generate different traction force directions depending on which teeth they are connected to [6].

According to the literature, the clinical success rate of miniscrew implantation exceeds 80%. Various factors influence this success rate, including bone quality, clinician expertise, smoking, oral hygiene, insertion torque, implantation site and angle, and proximity to tooth roots [7,8,9]. In addition to patient-specific factors, the clinician’s technique plays a critical role. Soft tissues in the oral cavity, including the cheeks, can impede the smooth manipulation of instruments, often preventing perpendicular insertion of the miniscrew into the bone surface [10,11].

Consequently, several studies have investigated the effects of oblique miniscrew insertion. For instance, Perillo et al. [12] reported that the optimal insertion angle should range between 0° and 60°, with different directions of applied force producing varying mechanical effects when screws are inserted obliquely. Similarly, Tatli et al. [13] analyzed the interaction of insertion angle and force direction, suggesting that angles greater than 60° may reduce miniscrew stability under multidirectional loading. Other studies using FEA, such as Cozzani et al. [14], have shown that the optimal insertion angle may vary depending on the miniscrew brand. Overall, these findings are inconsistent and warrant further investigation to determine whether variations arise from sample selection or incomplete experimental design.

Most previous studies, whether based on artificial bone experiments or FEA, have assumed that bone behaves as a homogeneous and isotropic material. However, from an anatomical and physiological perspective, bone exhibits direction-dependent mechanical properties [15,16]. Several studies have confirmed the mandible behaves as an orthotropic material [17,18]. Moreover, research in orthopedic trauma has compared isotropic and orthotropic bone properties, demonstrating that the material characteristics of bone significantly affect mechanical behavior [19,20,21].

In biomechanical analysis, FEA has been widely applied as an analytical method [22,23,24,25,26]. It allows simulation of complex bone structures and musculoskeletal loading systems. Furthermore, FEA enables the analysis of multiple models simultaneously, reducing experimental time, and provides detailed information on stress and strain distributions that cannot be obtained through conventional clinical or mechanical testing.

Despite substantial research on oblique miniscrew insertion and multidirectional loading, few studies have incorporated the orthotropic properties of bone into their analyses. Therefore, the extent to which orthotropic bone properties affect the finite element analysis results of miniscrews remains unclear in the current literature. The present study aims to address this gap by employing FEA to investigate the biomechanical responses of miniscrews inserted at various angles under multidirectional loading, comparing the outcomes between different mandibular bone models. Since clinicians insert miniscrews at different angles based on the planned direction of tooth movement, understanding from this study how mandibular bone anisotropy affects miniscrew performance can help clinicians avoid high-risk implantation angles and directions. This, in turn, has the potential to improve miniscrew success rates and provides significant clinical benefits.

## 2. Materials and Methods

The overall methodology involved creating a three-dimensional finite element representation of the miniscrew–bone system, assigning appropriate material properties, performing mesh generation and convergence testing, and applying clinically relevant loading conditions. Mechanical responses of the system were subsequently evaluated. Selected biomechanical parameters were used for this assessment.

### 2.1. Three-Dimensional Model Preparation

**Figure 1:**
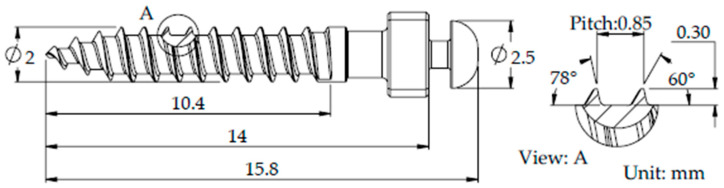
Schematic diagrams of the miniscrew.

A three-dimensional finite element model was established to investigate the biomechanical behavior of orthodontic miniscrews. The OBS miniscrew (model BD-2007-0, Bomei Co., Ltd., Taoyuan, Taiwan) was selected for analysis. This screw has a length of 12 mm and a diameter of 2.0 mm, with a thread depth of 0.3 mm and a thread pitch of 0.85 mm, as illustrated in Figure 1. The material of the screw was medical-grade stainless steel (SUS 316LVM, UNS S31673). All geometric models were constructed using SolidWorks 2017 (Dassault Systèmes SolidWorks Corp., Waltham, MA, USA).

**Figure 2:**
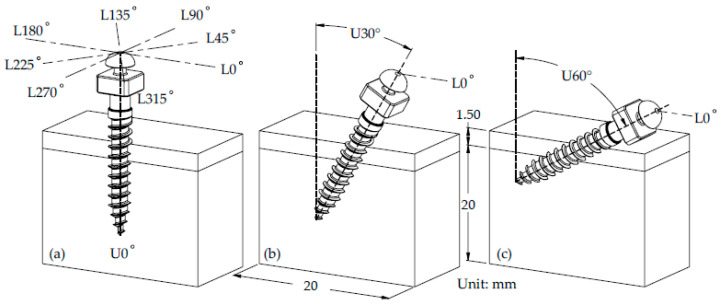
Schematic illustration of the miniscrew–bone model. (**a**) Definition of the loading direction; (**b**) definition of the oblique insertion angles; (**c**) definition of the bone block dimensions for analysis.

A simplified bone block model was constructed, consisting of cortical and cancellous bone layers. The block dimensions were set at 20 mm in length and width, with a cancellous bone height of 20 mm and a cortical bone thickness of 1.5 mm as shown in Figure 2b,c [27,28,29]. Due to the limitation of soft tissue interference, it was not feasible for the miniscrews to be fully embedded within the cortical bone under normal clinical conditions [30]. Therefore, the miniscrews were assumed to be inserted to a depth of approximately 8 mm, corresponding to 80% of the total threaded length, into the bone block. Three insertion angles were considered: 0°, 30°, and 60°, denoted as U0°, U30°, and U60°, respectively. The angle perpendicular to the cortical bone surface was defined as U0°, as illustrated in Figure 2a.

### 2.2. Material Property

An additional objective of this study was to investigate the influence of bone material properties on the biomechanical response of the miniscrew system. Accordingly, two types of material models were applied to represent cortical and cancellous bones. In the first model, both cortical and cancellous bones were assumed to exhibit isotropic, homogeneous, and linearly elastic behavior, as detailed in Table 1 [31]. In the second model, the bones were defined as orthotropic, homogeneous, and linearly elastic materials, with properties provided in Table 2 [17]. In all simulations, the miniscrew was composed of SUS 316LVM stainless steel, with its corresponding material properties also listed in Table 1 [32]. To simulate partial osseointegration [33], a fully bonded interface condition was applied by constraining the nodes at the miniscrew–bone interface [34].

**Figure 3:**
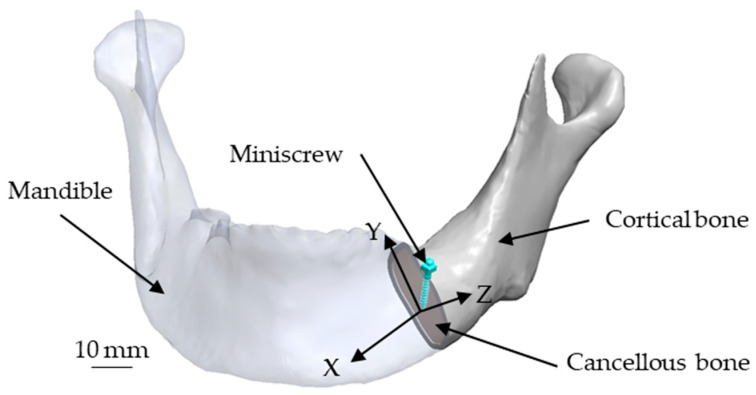
Definition of the coordinate system applied in this study.

According to the literature, the orthotropic material directions were defined as follows: the X-axis corresponded to the longitudinal axis, the Y-axis was perpendicular to the X-axis and parallel to the cortical bone surface, and the Z-axis was perpendicular to the XY-plane, as illustrated in Figure 3. In this study, the same coordinate system was applied to assign the material parameters in the analytical model.

### 2.3. Finite Element Analysis

**Figure 4:**
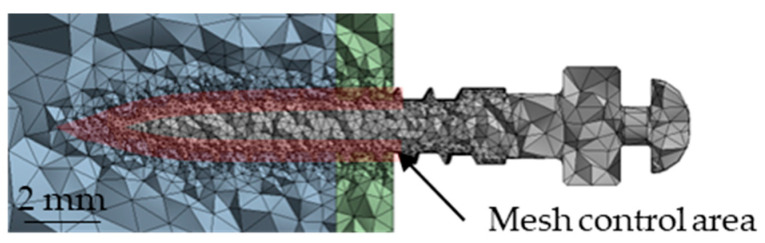
Definition of the mesh control area.

The bone block and miniscrew models were imported into ANSYS Workbench 2020R2 (ANSYS, Inc., Canonsburg, PA, USA) for finite element analysis. A 10-node tetrahedral mesh (SOLID187) was employed. A mesh convergence test was performed to ensure the accuracy and reliability of the simulation results. Several mesh element sizes ranging from 0.5 mm to 0.09 mm were assumed for comparison. The results obtained from FEA were evaluated using the selected indicators in this study. When the difference between successive mesh results was within 5%, the solution was considered converged. The smaller mesh size that achieved convergence was then adopted for subsequent analyses. The miniscrew–bone interface was assigned a refined mesh with a control size of 0.09 mm, as shown in Figure 4. Depending on the insertion angle, the total number of nodes ranged from 187,787 to 207,663, while the number of elements varied from 122,500 to 148,023, as shown in Table 3.

A loading force of 2 N, representing a typical orthodontic load applied in clinical practice [14,35], was applied to the cylindrical neck region of the miniscrew in eight directions: L0°, L45°, L90°, L135°, L180°, L225°, L270°, and L315°, as shown in Figure 2a. The applied force was defined as parallel to the cortical bone surface. To simulate boundary conditions, the bottom and lateral surfaces of the bone block were constrained.

### 2.4. Biomechanical Parameters for Evaluation

Mechanical responses were evaluated by predicting displacement, equivalent strain, von Mises stress, and principal stress at all nodes of the screw and bone structures. Particular emphasis was placed on the distribution of stress in the surrounding bone and on the screw surface, as well as the displacement at the load application point on the miniscrew head. In addition, the strain distribution within the bone block was regarded as a critical factor for assessing miniscrew stability and performance.

## 3. Results

### 3.1. Maximum Displacement in the Miniscrew and the Bone

**Figure 5:**
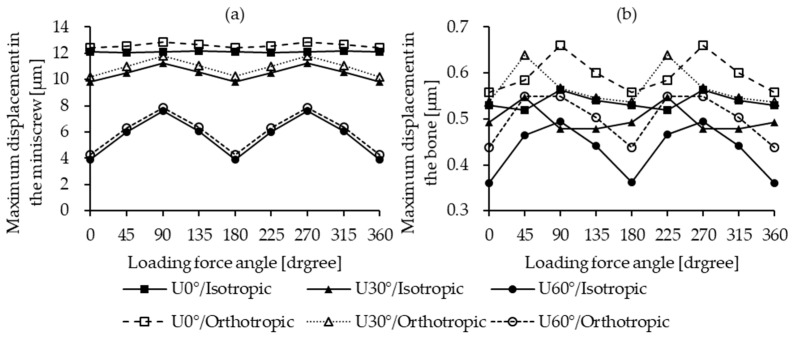
Maximum displacement under different conditions. (**a**) Miniscrew; (**b**) bone.

The maximum displacement of the miniscrew was observed in the U0° insertion model. This trend was consistent regardless of whether the bone material was modeled as isotropic or orthotropic. The displacement ranged between 12 µm and 13 µm. For the U30° and U60° insertion angles, the corresponding displacement ranges were 10–12 µm and 4–8 µm, respectively, as illustrated in Figure 5a. In contrast, bone displacement exhibited no significant variation across different insertion angles and remained minimal in all cases, ranging from 0.4 µm to 0.7 µm, as shown in Figure 5b.

### 3.2. Maximum Von Mises Stress in the Miniscrew

**Figure 6:**
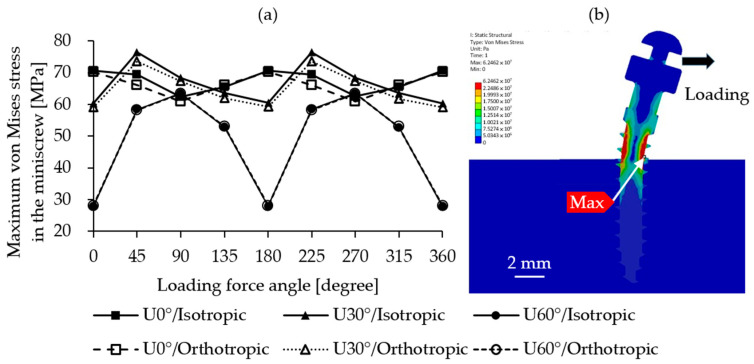
Von Mises stress distribution in the miniscrew under different conditions. (**a**) Maximum values; (**b**) stress distribution plot.

The von Mises stress experienced by the miniscrew showed negligible differences between the two mandibular bone models, with values remaining highly consistent. As illustrated in Figure 6a, the highest stress occurred under different loading directions depending on the insertion angle. For U0°, the maximum stress of 70.68 MPa was observed under the L180° loading direction. For U30°, the highest value was 76.48 MPa under L225°, and for U60°, the maximum stress was 63.44 MPa under L270°. In all cases, the stress concentration zones were located at the junction of the miniscrew and cortical bone on the tension side, as shown in Figure 6b. The U60° insertion model exhibited the greatest variation in von Mises stress across loading directions, with a difference of 35 MPa (63.44–27.88 MPa).

### 3.3. Maximum/Minimum Principal Stress in the Cortical Bone

**Figure 7:**
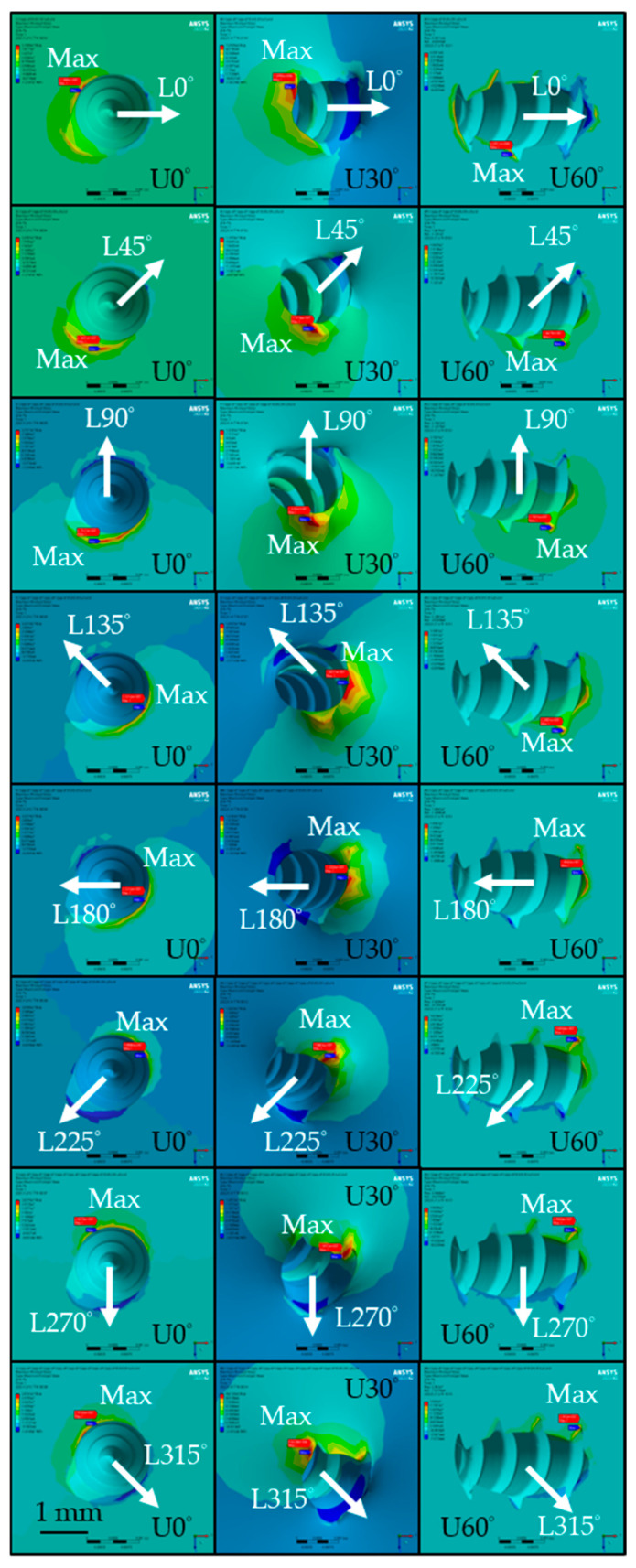
Distribution of maximum principal stress in the cortical bone under different conditions of the isotropic bone model. (The white arrows indicate the loading direction, U0°, U30°, and U60° represent the insertion angles, and the red labels mark the locations of the maximum values).

**Figure 8:**
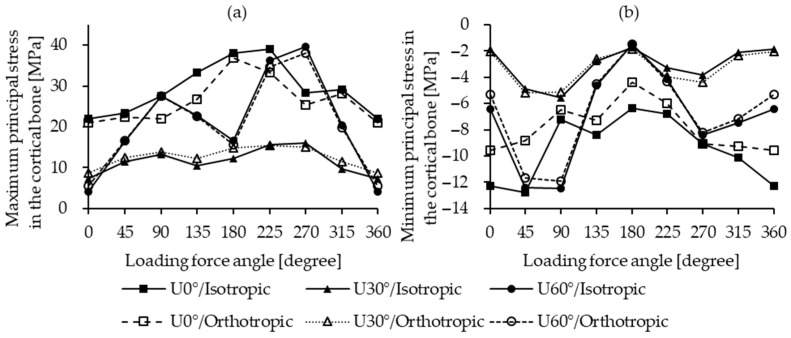
Principal stress in cortical bone. (**a**) Maximum principal stress; (**b**) minimum principal stress.

The distribution of maximum principal stress in cortical bone of the isotropic bone model is shown in Figure 7. The highest values for the U0° and U60° insertion angles were similar, reaching 38.98 MPa (L225°, isotropic) and 39.61 MPa (L270°, isotropic), as shown in Figure 8a, respectively. In contrast, the U30° model exhibited lower principal stress values across all loading directions, with a maximum of only 15.97 MPa under the L270° loading condition. Among the three angles, the U60° insertion demonstrated the greatest variation in principal stress due to different lateral loading directions, with a maximum difference of 35.51 MPa in the isotropic model, while U30° showed the smallest variation at 6.73 MPa in the orthotropic model.

**Figure 9:**
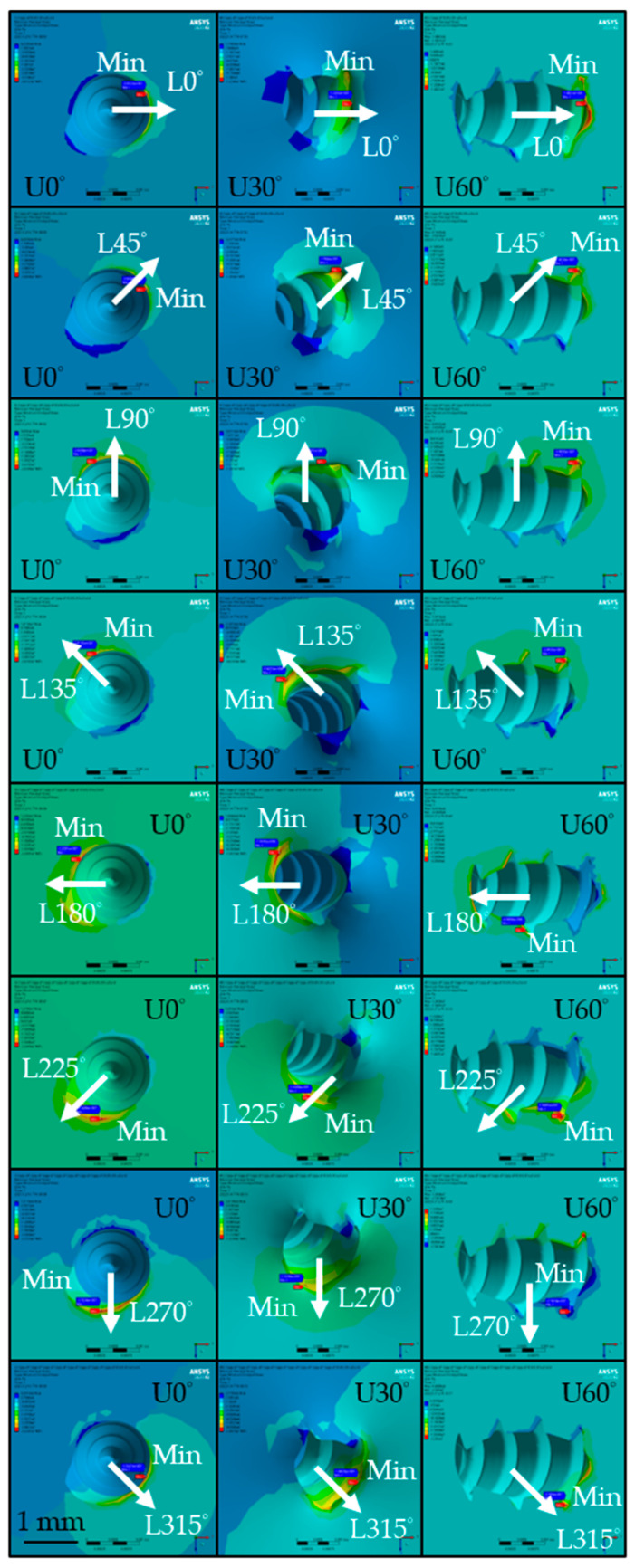
Distribution of minimum principal stress in the cortical bone under different conditions of the isotropic bone model. (The white arrows indicate the loading direction, U0°, U30°, and U60° represent the insertion angles, and the blue labels mark the locations of the minimum values).

Figure 9 shows the distribution of minimum principal stress in the cortical bone of the isotropic bone model. For the U0° and U60° models, the lowest stress values were −12.75 MPa (L45°, isotropic) and −12.48 MPa (L90°, isotropic), as shown in Figure 8b, respectively. For U30°, the lowest stress value was −5.53 MPa (L90°, isotropic), with values generally higher than those in the U0° and U60° models. The variation in minimum principal stress across different loading directions was largest for U60° (10.99 MPa, isotropic) and smallest for U30° (3.78 MPa, isotropic).

### 3.4. Maximum Strain in the Bone

**Figure 10:**
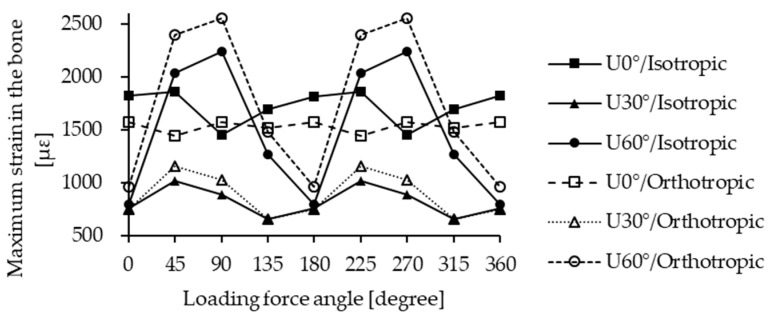
Maximum strain in bone under different conditions.

The maximum strain in the bone occurred predominantly within the cortical bone, as demonstrated in Figure 10. For the U0° and U30° insertion models, strain values did not vary significantly across loading directions. The maximum strains were 1820.2 µstrain under L0° loading (isotropic) for U0° and 1154.1 µstrain under L225° loading (orthotropic) for U30°. In contrast, the U60° insertion model demonstrated substantial variation in maximum strain across different loading directions, with a peak value of 2550.8 µstrain under L90° loading (orthotropic), representing approximately a 2.6-fold difference. For both U30° and U60°, the maximum strain values under each loading direction were generally higher in the orthotropic model compared to the isotropic one, except in the U0° model, where isotropic values exceeded orthotropic ones, except under the L90° and L270° directions.

## 4. Discussion

### 4.1. Anisotropic Bone Models

Regarding the maximum displacement in the miniscrew, values derived from orthotropic bone properties were generally higher than those from isotropic modeling. This trend aligns with previous literature findings [36,37]. The most significant differences were observed under the lateral loading directions of L90° and L270°, with percentage differences of 8.9% (U60°–L0°), 5.3% (U30°–L90°), and 6.1% (U0°–L90°), as shown in Table 4. From the perspective of miniscrew displacement, all percentage differences were positive, indicating that the orthotropic model produced larger displacement values. Since miniscrew displacement is often used as a key indicator for assessing primary stability [38,39], this finding suggests that isotropic modeling may lead to an overestimation of stability.

In terms of von Mises stress in the miniscrew, no significant difference was observed between the two mandibular bone models. This is because the maximum stress was located outside the bone rather than within it and therefore was not directly affected by the bone’s material properties.

For the maximum and minimum principal stresses in the cortical bone, the comparison between the two conditions revealed no definitive trend. The magnitudes varied without a consistent pattern. As presented in Table 4, the positive and negative values showed no regularity. This can be explained by the fact that stress concentrations were predominantly localized at the bone–screw interface, where both material anisotropy and geometric boundary effects exert a strong influence. Overall, the two models exhibited consistent trends in their responses under different loading directions.

### 4.2. Safety of Minscrew Design

The predicted maximum and minimum principal stress in the cortical bone ranged from 5.5 to 38.0 MPa and −12.7 to −1.74 MPa, respectively. These values are well below the reported yield strengths of cortical bone, which are approximately 100 MPa under tension and 150–200 MPa under compression [40]. Thus, the risk of microfracture under a 2 N orthodontic load is minimal, even for oblique insertion angles.

Similarly, the maximum von Mises stress observed in the miniscrew (76.48 MPa) remained below the yield strength of SUS 316LVM stainless steel, indicating that the current miniscrew design is mechanically safe under typical orthodontic forces.

It is noteworthy that the above results are based on the assumption of a fully bonded interface between the miniscrew and the bone. This condition represents the ideal state of complete osseointegration. However, before full osseointegration is achieved, part of the interface may still be in a mechanical anchoring phase characterized by nonlinear frictional contact. Therefore, during the stages prior to complete osseointegration, the present results may overestimate the stability of the orthodontic miniscrew. This is because the model does not account for the reduced stability associated with a non-osseointegrated interface.

### 4.3. Influence of Loading Direction and Insertion Angle

Under the U60° insertion condition (the steepest angle), variations in miniscrew maximum deformation and maximum von Mises stress became more pronounced, with the highest values observed under loading directions L90° and L270°. This discrepancy can be attributed to the inherent asymmetry of the screw thread geometry. When oblique insertion is unavoidable, minimizing miniscrew displacement and stress concentration can be achieved by aligning the loading direction as closely as possible with the plane parallel to the insertion angle. This finding can help clinicians reduce the risk of implantation failure caused by oblique insertion.

At the same loading direction, U0° insertion consistently resulted in the largest miniscrew displacement. According to existing literature, there is a negative correlation between miniscrew displacement and stability [41,42]. This outcome is attributed to the increased moment arm under lateral loading at a vertical insertion angle (U0°), which results in a larger bending moment.

When dividing the loading directions into two zones—Zone A (L0° to L180°) and Zone B (L180° to L360°)—the biomechanical indicators in both zones showed near-symmetrical behavior. This observation suggests that, despite the inherent asymmetry of the screw thread, the biomechanical response remains symmetric with respect to these zones. This finding is in agreement with prior research [43].

### 4.4. Strain Distribution in the Bone

A critical aspect of skeletal anchorage is the strain distribution in the surrounding bone, as strain is known to stimulate bone remodeling. The U0° insertion condition yielded broader and more uniformly distributed cortical bone strain, which may enhance osseointegration and promote bone regeneration, potentially improving stability. However, this benefit is accompanied by greater miniscrew displacement, which could negatively affect mechanical stability. These observations align with previous findings [44], which indicate that most stress is absorbed by the cortical bone due to its higher mechanical strength, rendering cancellous bone less significant in load-bearing.

In the U60° insertion condition, under L45°, L90°, L225°, and L270° loading directions, the maximum strain exceeded 2000 μstrain. According to the literature [8], bone strain levels between 1500 and 3000 μstrain are considered beneficial for remodeling, but excessive values within this range may paradoxically reduce osseointegration efficiency. Conversely, bone resorption may occur around the miniscrew, resulting in micromotion that compromises its stability and, in severe cases, triggering inflammatory responses due to extensive bone loss [33]. Therefore, a steep insertion angle such as U60° is not recommended. If such angulation cannot be avoided, loading forces in the L45° and L90° directions should be minimized to reduce the failure rate of the miniscrew.

### 4.5. Principal Stresses in the Cortical Bone

As cortical bone is a brittle material with higher compressive than tensile strength, maximum principal stress is a crucial factor influencing miniscrew stability [45,46]. As shown in Figure 7, the maximum principal stress (tensile side) in the cortical bone generally appeared on the side opposite to the loading direction. However, this trend was not consistently observed in the U60° insertion model, likely owing to the complex geometry at the bone–screw interface.

Unlike prior studies that assumed a sliding interface [43], the present study employed a fully bonded interface to simulate partial osseointegration, thereby restricting relative motion between bone and screw. This difference in boundary condition may explain the observed deviation in stress distribution.

In contrast, the minimum principal stress (compressive side) was consistently located on the same side as the loading direction, as shown in Figure 9, consistent with the previous findings [43]. This can be attributed to compressive forces stabilizing the bone–screw interface without inducing slippage. Furthermore, the predicted maximum principal stress did not exceed the reported yield strength of cortical bone (140 MPa) [18], confirming the mechanical safety of the current miniscrew design under a 2 N load.

## 5. Conclusions

Based on the finite element analysis results of this study and with reference to the relevant literature, the following conclusions are summarized. It should be noted that these conclusions are limited to finite element simulations and require further verification through clinical or experimental studies.

- Assuming isotropic bone properties leads to unrealistic predictions, underestimating miniscrew displacement and stability risk; orthotropic properties reflecting true bone anisotropy are therefore recommended.
- Increasing the miniscrew insertion angle reduces stability, particularly affecting bone strain and osseointegration potential, which vary with loading direction.
- Oblique miniscrew insertion may reduce stability, but aligning the load parallel to the insertion plane can mitigate adverse effects.
- For vertical insertion, thread design has minimal impact on displacement, von Mises stress, and bone strain; its influence increases at larger insertion angles.
- Certain biomechanical indicators (displacement, maximum von Mises stress, maximum strain) are symmetric across loading directions, allowing for an analysis of only half the directions.
- The current miniscrew design does not cause direct mechanical failure under clinical loading; most reported failures are likely due to biological factors, such as bone resorption or inflammation.

## 6. Limitations

In this study, the boundary condition between the bone and miniscrews was set as bonded, consistent with the partial osseointegration condition reported in the previous literature. However, this does not represent the only possible interaction. During the initial implantation phase, miniscrews may experience relative frictional sliding with the bone, a nonlinear behavior that is difficult to parameterize and varies widely in reported studies. Therefore, this study focused on the later stage of implantation, when partial bone integration has occurred, allowing for clearer interpretation of the observed effects. Consequently, the results reflect miniscrew behavior during the later phase of bone integration and cannot be generalized to the entire implantation process.

Alternatively, obtaining the elastic material properties of soft tissues and the nonlinear friction parameters at the miniscrew–bone interface from experimental studies or the literature could help address the limitations of the present study. This would allow for a more comprehensive analysis of the results.

## Figures and Tables

**Table 1:** Isotropic material properties of the miniscrew and bone.

Material	Young’s Modulus, E [MPa]	Poisson’s Ratio, ν
Cortical bone	13,700	0.3
Cancellous bone	1370	0.3
Miniscrew	200,000	0.33

**Table 2:** Orthotropic material properties of the bone.

Material	Young’s Modulus, E [MPa]			Poisson’s Ratio, ν			Shear Modulus, G [MPa]		
Direction	X	Y	Z	XY	YZ	XZ	XY	YZ	XZ
Cortical bone	19,600	13,800	10,600	0.38	0.23	0.47	6200	4100	5400
Cancellous bone	960	390	320	0.3	0.3	0.3	170	130	90

**Table 3:** Summary of node and element numbers for models with different insertion angles.

Insert Angle	Nodes	Elements
U0°	207,663	148,023
U30°	200,084	137,417
U60°	187,787	122,500

**Table 4:** Percentage difference between the two mandibular bone models.

	L0°	L45°	L90°	L135°	L180°	L225°	L270°	L315°	L360°
	Maximum displacement in the miniscrew [%]								
U0°	2.5	4.4	6.1	4.2	2.5	4.4	6.1	4.2	2.5
U30°	4.2	5.0	5.3	4.7	4.2	5.0	5.3	4.7	4.2
U60°	8.9	4.8	3.6	4.7	8.9	4.8	3.6	4.7	8.9
	Maximum von Mises stress in the miniscrew [%]								
U0°	−0.6	−4.7	−2.6	0.9	−0.6	−4.7	−2.6	0.9	−0.6
U30°	−2.1	−3.9	−1.5	−2.6	−2.1	−3.9	−1.5	−2.8	−2.1
U60°	0.4	−0.5	−0.1	0.3	0.4	−0.5	−0.1	0.3	0.4
	Maximum principal stress in the cortical bone [%]								
U0°	−4.8	−4.9	−20.1	−19.2	−3.1	−14.4	−10.1	−3.8	−4.8
U30°	19.8	9.4	5.6	16.6	21.5	−1.2	−5.7	18.5	19.8
U60°	34.3	1.2	−0.1	−1.0	−5.8	−4.6	−3.9	−2.9	34.3
	Minimum principal stress in the cortical bone [%]								
U0°	−22.2	−30.7	−10.8	−13.0	−31.0	−12.1	0.0	−8.8	−22.2
U30°	10.4	5.9	−7.3	−5.6	5.6	21.8	14.4	11.3	10.4
U60°	−16.8	−6.0	−4.7	−2.0	0.3	3.6	−2.4	−4.3	−16.8

Note: Difference (%) was calculated as (orthotropic − isotropic)/isotropic × 100%.

## Data Availability

The original contributions presented in this study are included in the article. Further inquiries can be directed to the corresponding author.
